# Solid Pseudopapillary Epithelial Neoplasm of the Pancreas in the Paediatric Population: A Report of Two Cases

**DOI:** 10.7759/cureus.29805

**Published:** 2022-10-01

**Authors:** Ravi Maharaj, Nahmorah J Bobb, Christo Cave, Keshan V Ramnarace, Jamar Critchlow

**Affiliations:** 1 Surgical Sciences, The University of the West Indies, St Augustine, TTO; 2 Clinical Surgical Sciences, The University of the West Indies, Champ Fleur, TTO; 3 Surgery, Eric Williams Medical Sciences Complex, Mt Hope, TTO; 4 Medicine and Surgery, The University of the West Indies, St Augustine, TTO; 5 Clinical Surgical Sciences, The University of the West Indies, Cave Hill, BRB

**Keywords:** spen, paediatric pancreatic neoplasm, paediatrics, abdominal mass, general surgery

## Abstract

A rare pathology, the solid pseudopapillary epithelial neoplasm (SPEN) of the pancreas accounts for approximately 1% of pancreatic neoplasms. Initially called ‘Frantz’s tumour’, it has now been renamed to SPEN by the World Health Organization (WHO). This tumour has a predilection for females and a good prognosis with surgical excision being the treatment of choice.

Palpable abdominal masses in children are of significant clinical importance. Identifying cystic lesions in the pancreas from CT or MRI scans always warrant further investigations. Primary pancreatic neoplasms account for 0.1% of pancreatic tumours in the paediatric population; an extremely rare circumstance constituting a diagnostic and therapeutic challenge to surgeons. This article comprises two paediatric cases of SPEN in 14- and 11-year-old females, respectively, and a literature review on current management.

## Introduction

A palpable mass in the abdomen of a child is a serious finding. Abdominal masses are most common in children under the age of five years, the majority arising from the kidney. When an older child presents with an abdominal mass it is more likely to be a malignancy. The most common tumours presenting as a large abdominal mass in an older child are Wilms’ tumour and neuroblastoma. The appearance of a cystic lesion in the pancreas on a computed tomography (CT) and magnetic resonance imaging (MRI) abdomen of a child being investigated for a symptomatic abdominal mass can cause a diagnostic dilemma.

The occurrence of primary pancreatic neoplasms in the paediatric population is exceedingly rare, comprising only 0.1% of pancreatic tumours from all age groups, and thus constitutes a diagnostic and therapeutic challenge to surgeons [1]. Such cases should be discussed at a multidisciplinary team meeting prior to any intervention such as a biopsy. Due to its rarity, the patient should be managed in a specialized centre with surgical oncologists who have experience in pancreatic resections for cystic neoplasms.

The solid pseudopapillary epithelial neoplasm (SPEN) of the pancreas is one of the rarest pathologies of the exocrine pancreas. Virginia Kneeland Frantz discovered this tumour and published her work in 1959 in a paper detailing its pathologic characteristics in three cases [2]. This tumour was subsequently renamed as a SPEN in 1966 by the World Health Organization (WHO). It is typically found in young females, almost ten times more common in females than in males [3]. This usually non-functional pancreatic tumour accounts for approximately 1% of all pancreatic neoplasms [4]. The SPEN tumour usually has a benign clinical course with a low potential for metastatic disease. The mainstay of treatment is total surgical extirpation with a cure rate of over 95%, with very few cases requiring adjuvant chemotherapy [5].

In the literature, there is a paucity of cases of SPEN in children compared to adults and thus we present two cases, a 14-year-old and an 11-year-old female, respectively, and a literature review on the current management with the emphasis on paediatric cases.

## Case presentation

Case report 1

A 14-year-old female with no prior medical conditions described intense abdominal pain after sustaining a fall during a sporting event. She described a central vague abdominal pain that was not relieved with the over-the-counter medication that her parents administered. Upon arrival at the emergency department, she had five episodes of bilious vomiting that had not happened before her admission to the hospital. The clinical examination revealed tenderness at the epigastrium and the right upper quadrant and had no evidence of peritonism.

**Figure 1:**
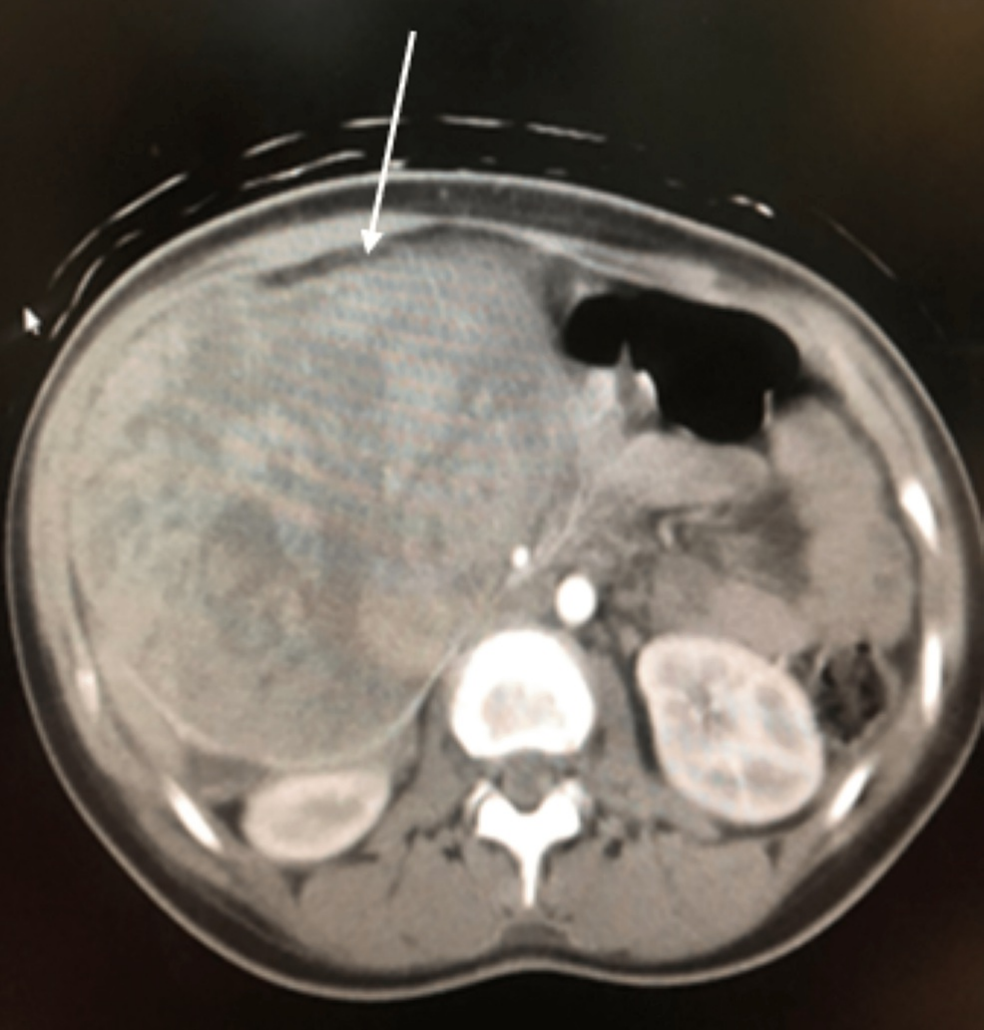
Single axial slice of the CECT demonstrating a larger heterogenous retroperitoneal mass (white arrow).

A round mass, firm in texture and fixed to the surrounding was present in the upper abdomen, measured approximately 8 cm x 8 cm, correlating with the tenderness found on clinical examination. Initial laboratory results were unremarkable, with adequate renal function and haematological parameters. Initial plain radiographs demonstrated no evidence of a pneumoperitoneum, nor was there any feature of intestinal obstruction. However, there was an unusual finding of bowel shadows isolated to the left side of the abdomen. Contrast-enhanced computed tomographic (CECT) imaging of the abdomen and pelvis revealed a pancreatic mass involving the body, neck, and head of the pancreas (Figure 1).

This lesion on CECT was heterogeneous, with multiple cystic components and areas of necrosis within a seemingly well-developed capsule. There was a definite mass effect from the tumour, with the displacement of the small bowel to the left lower abdomen. There was compression of the transverse colon, and the inferior vena cava (IVC) flattened in appearance. The lesion measured 11 cm (anteroposterior plane) x 14 cm (horizontal plane), and we noted an incidental finding of a replaced left hepatic artery. The discovery of this mass prompted a focused history of specific pancreatic exocrine and endocrine symptomatology. Our patient had no evidence of a functional pancreatic tumour on history or physical examination.

A multidisciplinary team meeting determined the next step in the management of the tumour, inclusive of a paediatric gastroenterologist, radiologists, oncologists, and senior surgical staff. During this discussion, preoperative histological sampling was a consideration, to confirm the diagnosis, however, the technology of endoscopic ultrasound (EUS) biopsy was not available. Additionally, the option of neoadjuvant chemotherapy was raised, in view to reduce the mass' size and reduce the possibility of anatomically contiguous 'en bloc' resections. The characteristics were, however, radiologically textbook for the identification of a SPEN. Most of the major vascular structures considered in pancreatic resections were all clear of the mass. There was some concern, however, about the relationship between the lesion, the splenic vein, the gastroduodenal artery, and the IVC.

**Figure 2:**
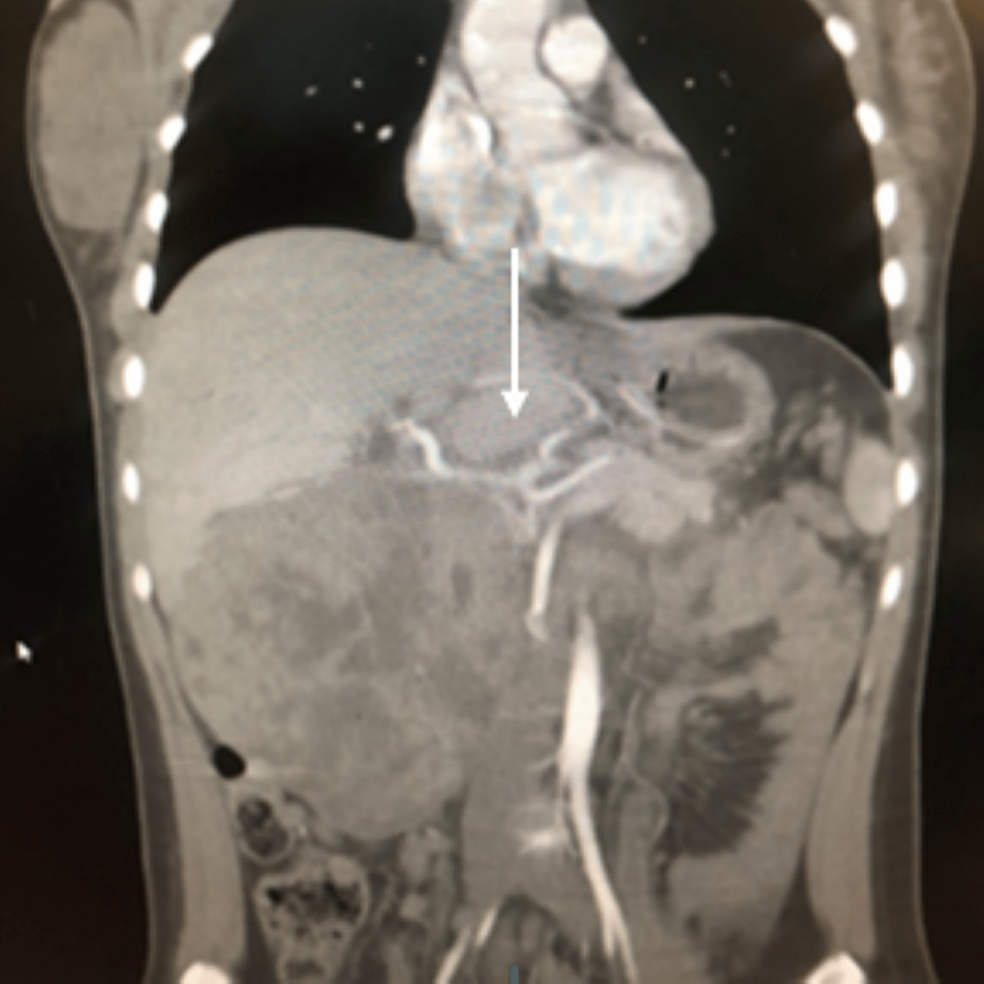
Single coronal slice of the retroperitoneal mass in addition to the presence of a replaced left hepatic artery arising from the left gastric artery (white arrow).

Interestingly, our patient also had a replaced left hepatic artery that originated from the left gastric artery before its anastomosing branches with the right gastric artery (Figure 2). The final decision from the meeting was conclusive that surgical extirpation, via a total pancreatectomy and splenectomy, was the definitive treatment and that preparation should be made for venous resection and reconstruction of the IVC if needed. Additionally, the patient was symptomatic as a result of the tumour’s compressive effect.

Team members were confident that radiologically, this was, in fact, a resectable SPEN, in correlation with its history and clinical behaviour. We chose a total pancreatectomy due to the prediction that the remnant pancreas, postoperatively, would not be sufficient to support physiological function. The possible involvement of the inferior mesenteric and splenic vein also lowered our threshold for a splenectomy. Her parents were counselled extensively on the decision made, and all this included the essential general and specific risks that may occur perioperatively. We chose an open approach, and intraoperatively, a detailed search for metastatic disease was performed. Our findings correlated with the CT findings of no gross haematogenous dissemination.

**Figure 3:**
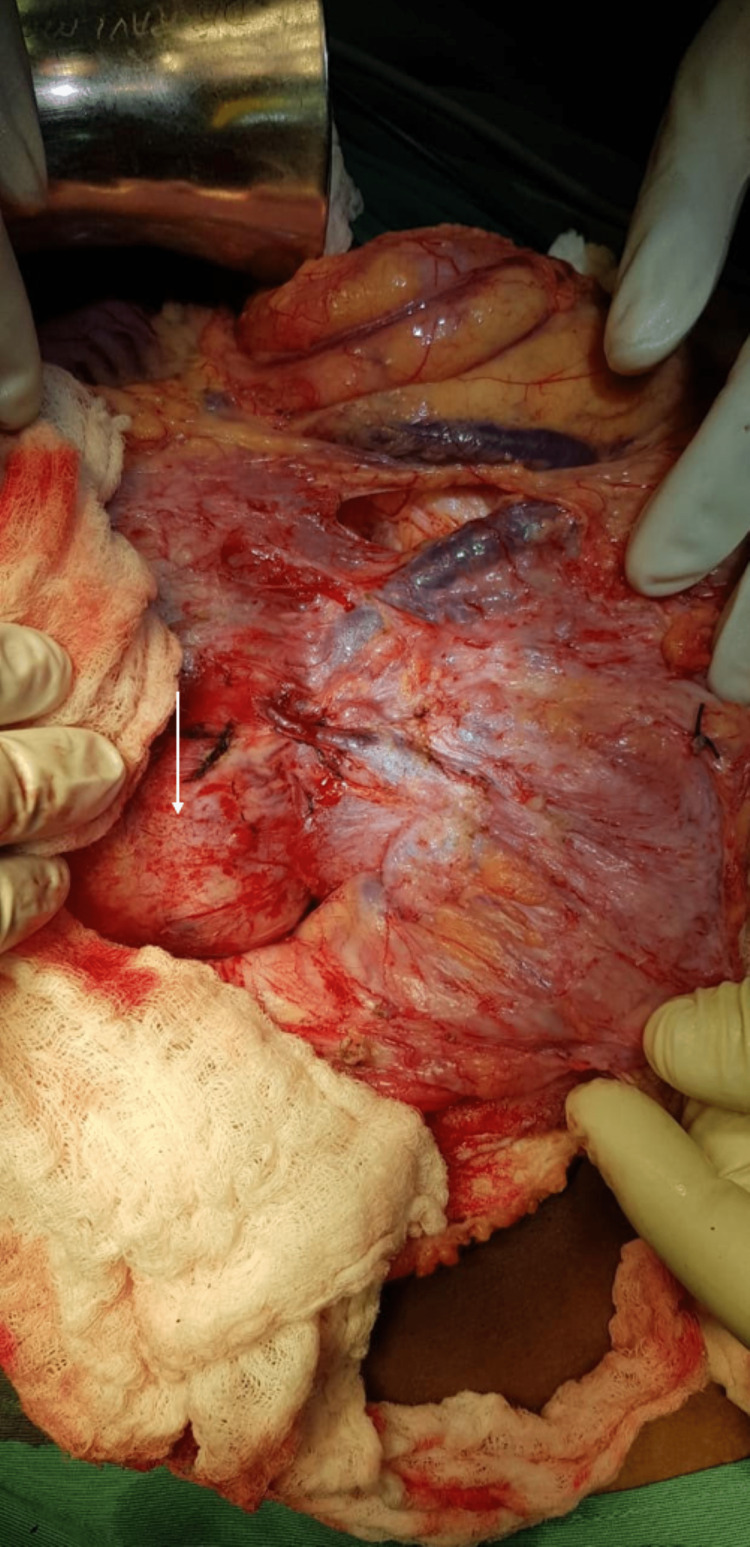
Demonstration of engorged mesocolic veins and impression of the retroperitoneal mass (white arrow).

The pancreatic mass involved the majority of the pancreas, and the remnant pancreas, if enucleation were chosen, would be technically challenging to reconstruct. It was inseparable from the right aspect of the mesocolon, splenic vein, and the inferior mesenteric vein (Figure 3). These findings suggested a more aggressive lesion, and our decision to perform an oncologic ‘en bloc’ resection was justified.

**Figure 4:**
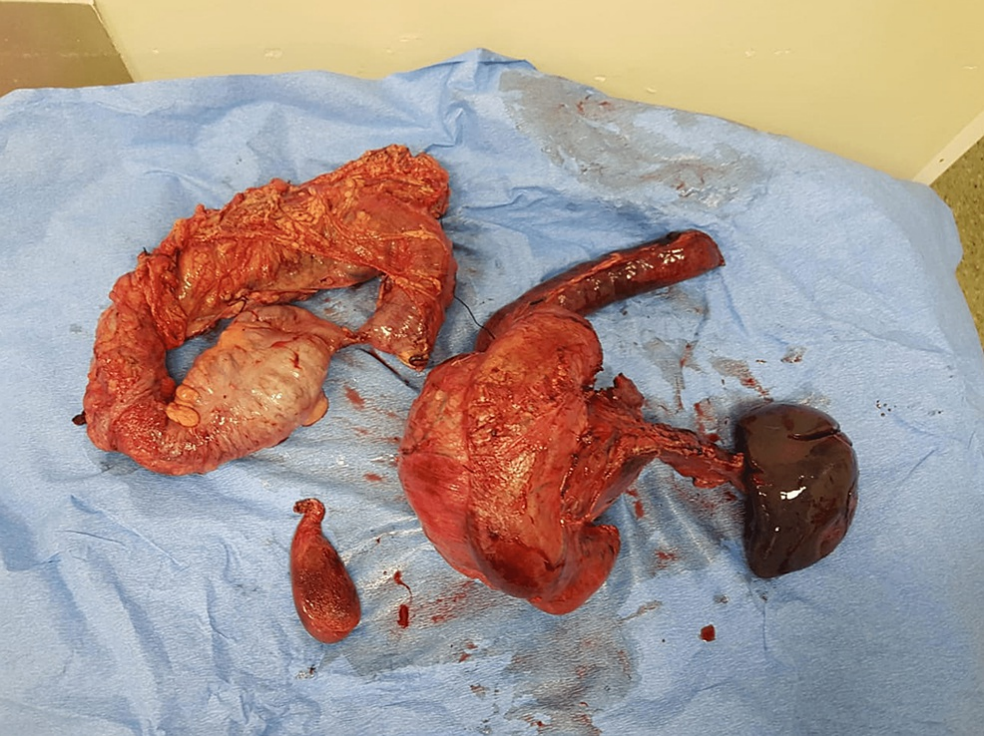
Depiction of the final resected specimen.

During the final stages of specimen removal, the tumour abutted the superior mesenteric vein, but not invaded by it. After the specimen was removed (Figure 4), the reconstructions included a hepaticojejunostomy in addition to a gastrojejunostomy. We chose a nasojejunostomy as the route of initial postoperative feeding, which we placed in the efferent limb of the gastrojejunostomy. Before the initiation of oral feeding, a water-soluble fluoroscopic study confirmed adequate flow and thereby patency of this anastomosis. The patient's nutrition was optimized with the help of the gastroenterologist in close communication with the dieticians, to manage her exocrine and endocrine pancreatic replacement.

Her recovery was uneventful and progressed without any degree of complications, and we discharged her to outpatient care. We received histological confirmation of a SPEN with no evidence of involved margins or capsular rupture. Additionally, there were no lymphatic metastases on the nodes harvested in the resection and no evidence of lymphovascular invasion. The specimen’s immunohistochemical panel was positive for neuron specific enolase as well as progesterone receptors (beta-catenin was not available in the immunohistochemical panel), in keeping with the diagnosis of a SPEN. She continued follow-up with the surgical team postoperatively in addition to a paediatric gastroenterologist as well as a paediatric endocrinologist. She is on pancreatic enzyme replacement currently and demonstrates no evidence of malabsorption such as diarrhoeal episodes, steatorrhea or nutritional deficiencies on serum evaluations. Initially, her glycaemic control postoperatively was challenging with both hyper- and hypoglycaemic episodes; however, this has been corrected with adjustments made by her endocrinologist.

Case report 2

An 11-year-old female was taken by her mother to a general hospital in south Trinidad, the San Fernando General Hospital (SFGH), for abdominal pain and abdominal distension occurring over six months. The abdominal distension was progressive, her abdomen became protuberant which prompted her mother to seek medical attention at SFGH. The abdominal pain was in the epigastrium, unrelenting, and had no radiation to other areas. It was associated with anorexia but no vomiting or constipation. She had no weight loss and there was no prior history of pancreatitis. An ultrasound was done in the accident and the emergency department showed an 8 cm intrabdominal mass that appeared to be arising from the liver.

The child was admitted to the paediatric ward for further investigation. A CT of the abdomen and pelvis with intravenous (IV) contrast was done which showed a 9 x 9 x 8 cm cystic mass in the distal pancreas. Subsequently, an open biopsy was done because the technology of EUS biopsy was not available. The histology reported a solid pseudopapillary neoplasm of the pancreas and the patient was transferred to a specialized paediatric oncology hospital.

**Figure 5:**
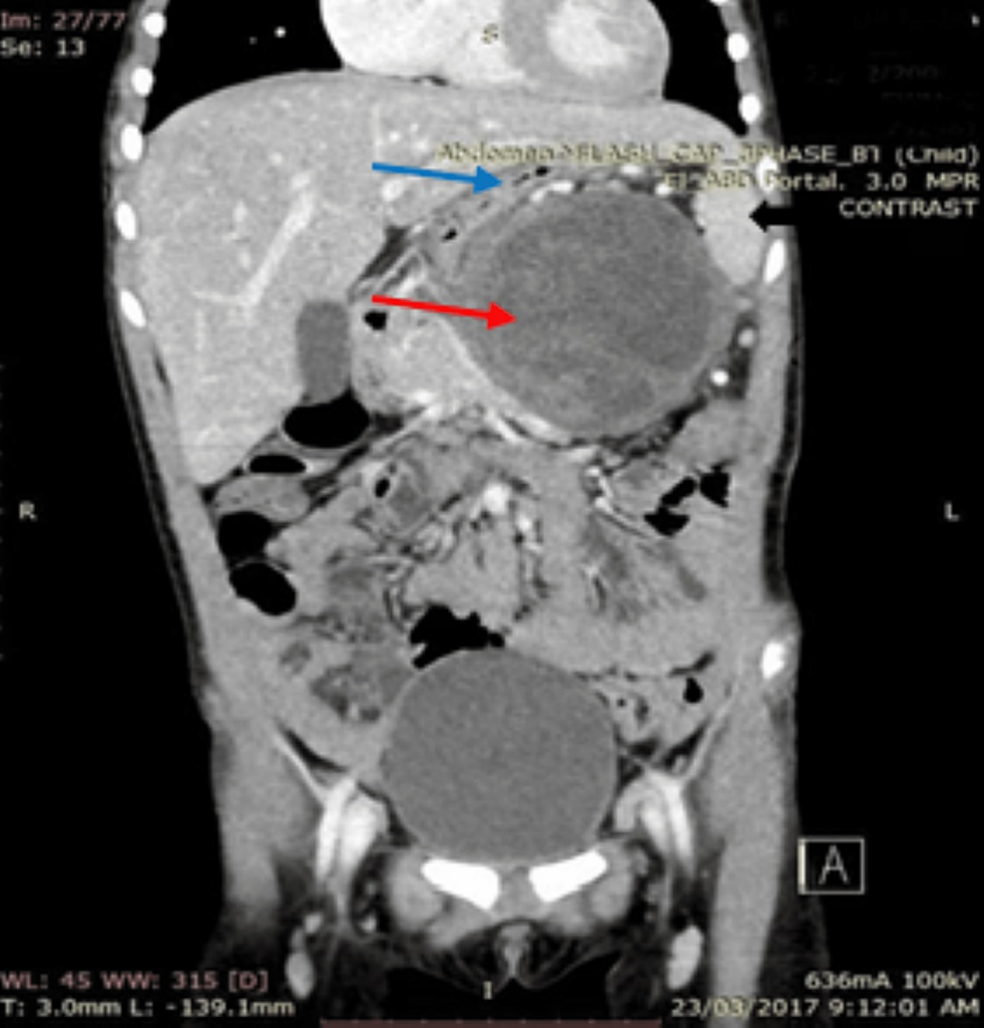
Cystic mass (red arrow) in the distal pancreas and its relationship to the spleen (black arrow) and the stomach (blue arrow).

**Figure 6:**
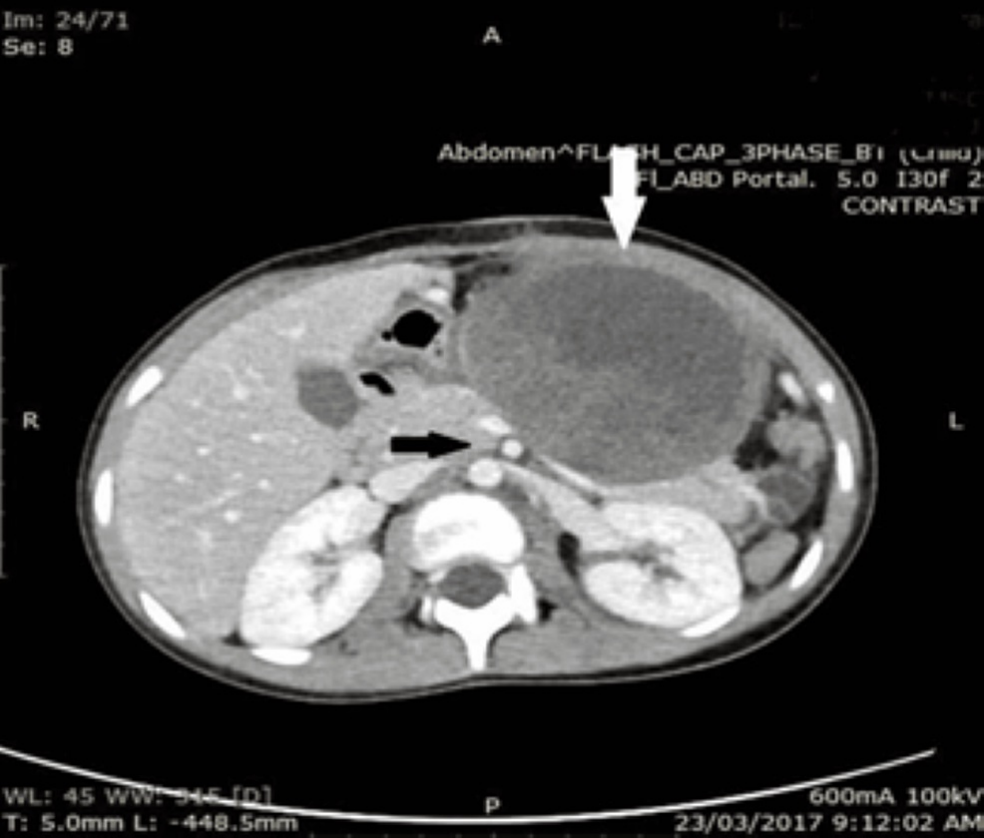
Pancreatic cystic mass (white arrow) arising from the body and tail of the pancreas. The SMV and SMA were not encased (black arrow).

A CT abdomen and pelvis were repeated and demonstrated an 8.7 x 8.4 x 7.2 cm cystic mass of mixed density (Figure 5), arising from the body and tail of the pancreas. The SPEN did not invade or encase the superior mesenteric artery (SMA) or the superior mesenteric vein (SMV) (Figure 6). Following a discussion in a multidisciplinary team meeting, an open distal pancreatectomy (ODP) and splenectomy were scheduled. The surgical oncology team consisted of paediatric and adult general surgeons. A chevron incision was made and the SPEN was found to be adherent to the left lobe of the liver, the stomach and the transverse colon. Adhesiolysis was performed and the greater omentum was divided to allow inferior displacement of the transverse colon.

The resection was performed using an antegrade approach. The pancreatic neck was transected with a linear stapler and the specimen was removed intact. Two 19 French abdominal drains were placed in the pancreatic bed and left upper quadrant respectively. A nasojejunal tube (NJT) was inserted and secured before abdominal closure. Postoperatively, the patient was diagnosed with a type B pancreatic fistula [6].

Elemental NJT feeds were administered, by day 10 post-op the child was ambulating and passing stool however a milky substance was seen emerging from the left upper quadrant drain. Further biochemical analysis confirmed there was a chyle leak which was managed conservatively. An abdominal US revealed no intra-abdominal fluid collections. Streptococcus pneumoniae, haemophilus influenzae type B and neisseria meningitidis vaccines were administered approximately 14 days postoperatively. On day 22, the pancreatic fistula resolved, and the patient was discharged to the outpatient clinic. She had an abdominal ultrasound 30 days after the resection of the SPEN which reported the proximal pancreas appeared normal with no peripancreatic fluid collection.

**Figure 7:**
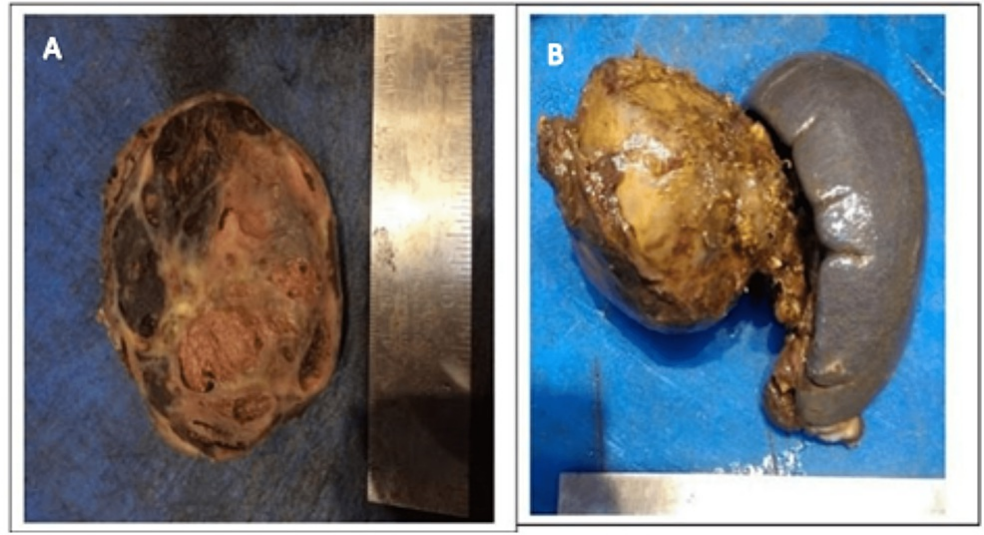
(A, B) Gross histology specimen showing the variegated solid and cystic appearance with thromboses.

The final histopathology report stated the specimen was an 8.5 cm solid pseudopapillary neoplasm in the tail of the pancreas. The surgical margins were negative for tumour and three lymph nodes resected did not have tumour involvement. The cut surface of the tumour had a variegated solid and cystic appearance with thromboses and haemorrhagic regions (Figures 7A, 7B). The immunohistochemical analysis was positive for vimentin, alpha-1-antitrypsin and neuron specific enolase, spotted expressed chromogranin and synaptophysin. At one-year follow-up, an MRI of the abdomen and pelvis was performed which did not show any recurrence of the tumour. She is currently in good health and awaiting her two-year surveillance scan.

## Discussion

The SPEN of the pancreas is an uncommon tumour of its exocrine component. Historically, Moynihan and Robson documented cystic neoplasms of the pancreas in separate instances in 1903, before Dr. Frantz's description [7]. Kloppel et al., in conjunction with the World Health Organization, renamed this tumour to what we know it to be today. They also defined it as a 'low-grade carcinoma composed of monomorphous cells forming solid and pseudopapillary structures' [8]. Its documentation in the literature has increased gradually over the recent decades, with initially fewer than 50 cases in the 1970s to 130 cases described in 1992 by Papavramidis and Papavramidis [9].

The name encompasses two distinctive features seen in histology, solid and pseudopapillary areas. On gross examination, large cystic or cystic and solid tumours with necrotic and haemorrhagic zones are seen. The origin of these solid pseudopapillary tumours is not clear, one theory is that they arise from the multipotent primordial cell while another suggests it originates from genital ridge angle-related cells [9].

Currently, the literature comprises numbers over 2,500 cases, evidenced by an extensive systematic review done by Law et al. at the Johns Hopkins School of Medicine [10]. Locally, our data demonstrate an incidence of 0.17 per 100,000, with 11 cases identified from June 2012 to July 2017. These cases included only females, and the average age of diagnosis was 25.78 years +/- 7.8 years, and almost two-thirds of this population were of Afro-Caribbean descent [11]. As with our local data, the most commonly recognized age range falls between 20 and 30 years old [9,10].

The most common clinical finding of these lesions includes vague abdominal pain, with one review demonstrating 63% of patients describing this as the only symptom present at the time of diagnosis [10]. Alternatively, the SPEN is also detected as an asymptomatic mass after a routine examination or an incidental finding after accidental injury, as happened with our case [7]. Mass effect causing compression on the surrounding small intestine has also been documented, and symptoms of early satiety and nausea with vomiting are described additionally [12]. Interestingly, masses located at the head of the pancreas, irrespective of their size, rarely obstruct the extrahepatic biliary tree [9]. Rarely, these tumours have ruptured their capsules and presented with signs of an acute hemoperitoneum such as tenderness and peritonism [13].

Differentiation from other cystic neoplasms, in the paediatric population, is necessary. These include pathologies such as a pancreatoblastoma in younger patients, a pancreatic pseudocyst, and secondary pancreatic tumours, for example, in lymphoproliferative disorders and neuroblastoma [12]. Various imaging investigations are utilized in the workup of this tumour, such as ultrasonography, CT as well as MRI. Heterogenous, well-defined hypoechoic solid masses that may or may not possess interspersed cystic areas are noted on ultrasound assessment. These findings may be accompanied by semi-circular calcifications, internal septae as well as displacement of surrounding viscera [14].

The CT features suggestive of a SPEN are a well-encapsulated or partially encapsulated heterogeneous lesion in the pancreas with scattered areas of haemorrhage, necrosis, and cystic degeneration. Enhancement on the CT usually remains isodense amongst the arterial and venous phases, which is a differentiating feature from pancreatic neuroendocrine tumours which enhance significantly in the arterial phase and wash out quickly in the venous phase [15]. CT delineates anatomical relationships between the tumour and surrounding structures with exceptional spatial resolution [16]. Calcifications may also be seen on CT, and they usually are within areas of degeneration, in keeping with dystrophic calcification, close to areas of necrosis, as opposed to a microcystic adenoma, which demonstrates a 'sunburst' pattern of calcification [17].

The use of an MRI abdomen can aid with differentiating a SPEN from other tumours with its high degree of soft tissue characterization, assessing areas of haemorrhage and necrosis, for example. The apparent diffusion coefficient is another method on MRI to help distinguish the SPEN from other differentials, as this value is much higher than other cystic lesions of the pancreas. This measurement depends on the extent of tissue cellularity and water diffusion [15]. Angiographically, the SPEN is relatively hypo-vascular, even avascular in some cases, which also assists in the exclusion of other differential diagnoses [18].

A pre-operative diagnosis can be made with a biopsy; however, CT-guided biopsy is not recommended because it can cause rupture of the tumour, haemorrhage and seeding of the tumour by peritoneal or cutaneous contamination during sampling [19]. A laparoscopic biopsy is also not advised due to its invasive nature and similar risks. A definitive diagnosis can be made using EUS and fine needle aspiration (FNA). EUS-guided FNA is recommended in the diagnostic workup of a suspected pancreatic neoplasm by the American Gastroenterology Association and the 2018 European Guidelines on pancreatic cystic neoplasms [11,20-22]. EUS is not available in our region, and this played an important role in the decision made at the multi-disciplinary meeting.

EUS also provides additional information necessary in planning a surgical resection such as the exact size of the tumour, local invasion, associated lymph nodes and hepatic metastases. The location of the lesion will determine the route of FNA, a trans gastric approach is adopted for lesions in the body or tail of the pancreas whereas tumours in the head or uncinate process are aspirated via the duodenum, this facilitates a short needle tract. On EUS, a solid pseudopapillary tumour will have the characteristic appearance of a heterogeneous solid, mixed solid, or cystic hypoechoic lesion [23]. The potential for needle tract seeding is negligible with FNA using EUS because of the short needle path [24]. The technique has been successfully performed in children with SPEN [10,25-28]. Another distinct feature of these tumours is the presence of progesterone receptors and oestrogen receptors, the former being present in up to 90% of tumours [29].

A systematic review has reported that cytology of EUS aspirate has good specificity for pancreatic cancer of 90.6%, but a low sensitivity of 64.8% [30]. One reason for the poor sensitivity is the low cellularity of cytology samples, especially in pancreatic cystic lesions. It has a diagnostic accuracy of 75%-100% for SPEN based on the case series published in the literature for SPEN [10,23,26,27,31]. The cytopathology is characteristic, branching papillae with a central vascular core and surrounding myxoid stroma [23,25].

The diagnosis can be confirmed by performing immunohistochemistry. Solid pseudopapillary neoplasms show immunoreactivity to vimentin, alpha-1-antitrypsin, phospholipase A2, beta-catenin, alpha-1-antichymotrypsin, CD10 and neuron-specific enolase [25,29,32,33]. The use of tumour markers does not usually contribute to the establishment of the diagnosis. The commonly associated hepato-bilio-pancreatic markers, such as carbohydrate antigen (CA) 19-9, alpha-fetoprotein (AFP), and carcinoembryonic antigen (CEA), are not elevated in this disease where high levels of CEA in the cyst fluid (>192ng/mL) is more suggestive of a mucin-producing neoplasm such as IMPN rather than a SPEN [12,34].

Aggressive resection of SPEN tumours results in loss of pancreatic parenchyma and may lead to pancreatic insufficiency in children. This has been a major concern of pancreatic resections and a more conservative approach has been advocated by performing enucleation of the tumour instead. Solid pseudopapillary neoplasms are surrounded by a fibrous capsule that can be excised from the adjacent parenchyma without removing pancreatic tissue. Tumour involvement of the main pancreatic duct, local invasion into the pancreas or surrounding organs and metastases are contraindications to the use of this technique. The use of intraoperative frozen section should be employed to ensure the surgical margin is free of tumour and thus confirm a complete resection. The presence of positive margins and suspicion of malignancy mandates surgical resection and removal of the distal or proximal pancreas depending on tumour location. 

Wang et al. published the retrospective series with the largest number of SPEN cases that were treated with enucleation [35]. It included 110 patients, consisting of adults and children, who were treated at a single institution between 2009 and 2016. Enucleation was performed in 31 patients; they were compared to 70 patients that had a conventional pancreatic resection. The most common complication post enucleation was post-operative pancreatic fistula and there were no recurrences after 46.1 months of follow-up. The operating time was significantly reduced in the enucleation group (155 vs 245 minutes), also the blood loss was reduced (140 vs. 380 mL). The morbidity was similar between the two groups 25.8% vs 24.3% in the enucleation group and the pancreatic resection group respectively. Concerning pancreatic function, there was a statistically significant lower rate of exocrine and endocrine insufficiency in the enucleation group.

Despite the potential for preservation of pancreatic function and the lower morbidity associated with enucleation, the majority of cases of SPEN reported have been treated with pancreatic resection. The proponents of aggressive surgical resection have stated that the tumour has malignant potential and thus should be treated with wide resection. Other arguments include, it is not rare that the tumour partially lacks a capsule in areas of local infiltration, the tumour occasionally incorporates the main pancreatic duct into the capsule wall and metastasizing SPEN shows a low growth fraction [36]. In addition, dissecting along the tumour capsule wall during enucleation may leave positive margins, and local recurrence has been reported. It is difficult to assess surgical margins accurately with by frozen section biopsy [36]. 

A meta-analysis done in 2015 by Hüttner et al. collected data from 22 observational studies, nine of which were prospectively executed, including over 1,100 patients. The startling difference between these resections was the higher rate of postoperative pancreatic fistulae (POPF) that occurred with enucleation, 25.5% versus 19.7%. These leaks were not found to impact overall morbidity or mortality. Additionally, this meta-analysis included cystic neoplasms of the pancreas in addition to the SPEN; hence, it may be a confounding variable in the assessment of enucleation strictly in cases of SPEN. However, the benefits of less functional disruption, shorter operative times, and less intraoperative blood loss were retained, with no differences in overall mortality calculated. One of the major critiques in this study consisted of the failure of addressing the comparative results of disease-free survival between the arms of treatment, as most of the studies covered short-term parameters [37].

Surgical options, concerning anatomical resections, depend on the location of the tumour, as well as its relation to important vaso-biliary structures related to the pancreas. Lesions in the head or uncinate regions of the pancreas benefit from pancreaticoduodenectomy with pancreatic reconstruction via jejunal or gastric anastomotic outlets. Tumours at the neck of the pancreas are dealt with via central pancreatectomy, which includes a distal pancreaticogastrostomy and over-sewing of the proximal remnant. Its drawback comes from two sources of pancreatic leakage postoperatively and is advised to be done only by extensively trained and experienced in pancreatic resections [38].

Body and tail lesions are effectively resected via distal pancreatectomy. Splenic preservation has been debated in these tumours as the argument of adequate lymphadenectomy holds little support in SPEN cases. Prophylactic lymphadenectomy is not usually required, considering the incidence of lymph node involvement is rare [10]. Exceptions are made, however, in cases where the splenic artery or vein is involved in the neoplastic process or if there is significant splenomegaly with no other attributable cause [39]. An important operative principle during these resections, whether enucleation or anatomic, is to avoid capsular rupture and its risk of peritoneal carcinomatosis [40].

The guidelines for the management of SPEN were produced mainly from adult cases of SPEN. Bender et al. published the only systematic review of SPEN in the paediatric population in 2018 [28]. They reviewed 135 articles; 70% of which were case reports. There were no randomized control trials. The review included 523 paediatric patients, 83% of whom were female. Most of the SPEN were in the pancreatic tail, 54%, with the other 46% confined to the pancreatic head. A preoperative biopsy was performed in only 16.7% of cases, 6.7% had a traditional open biopsy and 10% were subjected to FNA. Five hundred and seven patients (96.9%) underwent surgical resection. 

The Whipple’s procedure and distal pancreatectomy were the most frequent operations performed at 28.6% and 26.8%, respectively. A smaller group of patients had distal pancreatectomy and splenectomy (14.2%), the lesser common operations were enucleation (6.5%), central pancreatectomy (6.3%) and head resection (4.1%). In some patients the operative procedure performed was not specified (13.4%). The development of a pancreatic fistula was the most frequent complication reported. The other complications were delayed gastric emptying, pancreatic leak, pseudocyst formation, bleeding and pancreatitis. 

The prognosis of SPEN in children is good. Four hundred (96%) of the patients were successfully followed up for 46.6 months. The majority, 93.3%, remained disease free and only 3.8% required adjuvant therapy. There were no deaths recorded in any of the studies, however, 6.7% had recurrent disease. In comparison with the data from adult studies, SPEN is still more prevalent in the female sex and more frequently located in the tail. In addition, surgery was also the mainstay of therapy. 

Laparoscopic distal pancreatectomy (LDP) has been performed safely in several case series of SPEN in paediatric patients [41-43]. Relative contraindications to its use include large SPEN tumours (>7cm), a hostile abdomen and a lack of laparoscopic experience. Splenic preservation can be achieved in LDP. A retrospective study compared LDP to open distal pancreatectomy (ODP) [44]. Fourteen patients who had LDP were compared to eight patients having undergone ODP. The length of operating time was significantly reduced in the LDP group compared to the ODP group (175 vs 257 minutes) [44]. The postoperative complication rate was the same in both groups. However, the LDP patients resumed oral intake earlier than the ODP patients (p = 0.010), and had a shorter hospital stay (p = 0.009) [44]. The use of laparoscopy in pancreatic resections of SPEN is fairly new and limited to a small number of case series, larger case series or systematic reviews are required to determine if it should be the standard of care.

Clinicopathological features that may be predictive of malignant change and disseminated disease include male gender, tumours over 5cm, capsular rupture, and high mitotic counts [45]. Even in the presence of liver, and even peritoneal metastases, targeted therapy achieves a high rate of cure. The peritoneal disease has been effectively treated with complete cytoreductive surgery in combination with hyperthermic intraperitoneal chemotherapy with agents such as irinotecan [46]. Options for hepatic metastases include radiofrequency ablation, trans-arterial chemoembolization, and even liver transplantation, all associated with an increased survival benefit [47,48]. Malignant variants occur in approximately 18% of cases in the adult demographic. However, there is very little standardization in the criteria for malignancy [49].

Butte and colleagues outlined that SPEN may be defined as malignant in cases of recurrence, distant metastases, or an unresectable tumour with macrovascular invasion [49,50]. Chemotherapy is a difficult decision to make as a result of a lack of data on its usage, and data that is available originates from smaller case reports and series. Gemcitabine is among the first choices for chemotherapy in aggressive variants of this disease [51]. Ji et al. investigated the use of neoadjuvant chemotherapy in the case of a 57-year-old woman with unresectable disease. They noted evidence of tumour regression, and subsequent resection, resulting in advocating its purpose in usage in unresectable [52].

## Conclusions

SPEN of the pancreas is a rare constituent amongst cystic pancreatic neoplasms with a definite predilection for younger females. Clinical suspicion of this tumour in an adolescent female with an upper abdominal mass devoid of exo- or endocrinopathy is paramount. Its indolent nature and its low metastatic potential make it a candidate for aggressive surgical resection, which results in very favourable disease-free survival and overall survival rates.
